# Endovascular Treatment of Visceral Artery Pseudoaneurysms with Ethylene-Vinyl Alcohol (EVOH) Copolymer-Based Non-Adhesive Liquid Embolic Agents (NALEAs)

**DOI:** 10.3390/medicina59091606

**Published:** 2023-09-06

**Authors:** Roberto Minici, Pasquale Guerriero, Federico Fontana, Massimo Venturini, Giuseppe Guzzardi, Filippo Piacentino, Andrea Coppola, Marco Spinetta, Agostino Siciliano, Raffaele Serra, Davide Costa, Nicola Ielapi, Rita Santoro, on behalf of the MGJR Research Team, Luca Brunese, Domenico Laganà

**Affiliations:** 1 Radiology Unit, Dulbecco University Hospital, 88100 Catanzaro, Italy; miniciroberto@gmail.com (R.M.); agostino.siciliano@tiscali.it (A.S.); domenico.lagana@unicz.it (D.L.); 2 Department of Medicine and Health Sciences, University of Molise, 86100 Campobasso, Italy; luca.brunese@unimol.it; 3 Diagnostic and Interventional Radiology Unit, ASST Settelaghi, Insubria University, 21100 Varese, Italy; federico.fontana@uninsubria.it (F.F.); massimo.venturini@uninsubria.it (M.V.); filippo.piacentino@asst-settelaghi.it (F.P.); andrea.coppola@asst-settelaghi.it (A.C.); 4 Radiology Unit, Maggiore della Carità University Hospital, 28100 Novara, Italy; giuguzzardi@gmail.com (G.G.); marcospinetta90@gmail.com (M.S.); 5 Vascular Surgery Unit, Department of Medical and Surgical Sciences, Magna Graecia University of Catanzaro, Dulbecco University Hospital, 88100 Catanzaro, Italy; rserra@unicz.it; 6 Department of Law, Economics and Sociology, Magna Graecia University of Catanzaro, 88100 Catanzaro, Italy; davide.costa@unicz.it; 7 Department of Public Health and Infectious Disease, Sapienza University of Rome, 00185 Rome, Italy; nicola.ielapi@uniroma1.it; 8 Haemophilia and Thrombosis Center, Dulbecco University Hospital, 88100 Catanzaro, Italy; ritacarlottasantoro@gmail.com; 9 Magna Graecia Junior Radiologists Research Team, 88100 Catanzaro, Italy; radiologyumg@gmail.com

**Keywords:** pseudoaneurysm, visceral pseudoaneurysms, transcatheter arterial embolization, embolization, endovascular treatment, percutaneous, embolic agents, Ethylene-Vinyl Alcohol, non-adhesive liquid embolic agents, Onyx

## Abstract

*Background and Objectives*: Treatment of visceral artery pseudoaneurysms (VAPs) is always indicated regardless of their diameters, as their risk of rupture is significantly higher than that of visceral artery aneurysms. The invasiveness of surgery and its associated complications have led to a shift in favor of radiological interventions as the initial treatment of choice. However, there are still some unanswered questions on endovascular treatment of VAPs regarding the optimal endovascular technique and the efficacy and safety outcomes. The purpose of this multicenter study was to retrospectively evaluate the effectiveness and safety of endovascular treatment of visceral pseudoaneurysms using Ethylene-Vinyl Alcohol (EVOH) Copolymer-Based Non-Adhesive Liquid Embolic Agents (NALEAs). *Materials and Methods*: Consecutive patients who underwent endovascular embolization with EVOH-based NALEAs for visceral artery pseudoaneurysms between January 2018 and June 2023 were retrospectively evaluated. *Results*: 38 embolizations were performed. Technical success was achieved in all patients. The clinical success rate was high (92.1% overall), with no significant differences between ruptured and unruptured VAPs (*p* = 0.679). Seven patients (18.4%) experienced procedure-related complications, related to one case of non-target embolization, four splenic abscesses due to end-organ infarction, and two femoral pseudoaneurysms. The rates of procedure-related complications, end-organ infarction, and vascular access-site complications did not significantly differ between ruptured and unruptured VAPs (*p* > 0.05). *Conclusions*: Both ruptured and unruptured visceral pseudoaneurysms can be effectively and safely treated with NALEA-based endovascular embolization. We suggest considering the use of NALEAs, particularly in specific clinical cases that highlight their advantages, including patients with coagulopathy, fragile vessels, and embolization targets that are located at a considerable distance from the microcatheter tip and are otherwise difficult to reach.

## 1. Introduction

Visceral artery pseudoaneurysms (VAPs) are critical vascular abnormalities originating from the splanchnic circulation, often attributed to inflammatory, infectious, traumatic, iatrogenic, and neoplastic etiologies [1,2,3,4,5,6,7,8,9,10,11,12,13]. Distinct from true aneurysms, pseudoaneurysms arise from the disruption of the intimal and medial layers of the arterial wall, lacking an epithelized wall structure [14]. Pseudoaneurysms pose a significant risk to patient health and survival [1,2].

Treatment of VAPs is always indicated regardless of their diameters, as their risk of rupture is significantly higher than that of visceral artery aneurysms (VAAs) [15,16,17]. VAPs ruptured at a substantially greater incidence than VAAs (76.3% vs. 3.1%), according to an investigation of 233 individuals by Pitton et al. [18]. Visceral pseudoaneurysms with and without rupture did not differ significantly in size, and the rupture event is not reliably predicted by diameter [18]. Compared to 30% of VAAs, 80% of VAPs were symptomatic upon presentation according to a series by Tulsyan et al. [19]. Therefore, treatment of VAPs is recommended as soon as possible [17].

Traditionally, surgical intervention has been the primary approach for addressing pseudoaneurysms [15,17]. Nevertheless, the invasiveness of surgery and its associated complications have led to a shift in favor of endovascular interventions as the initial treatment of choice. Endovascular interventions offer the advantages of minimally invasive procedures, demonstrating notable success rates and minimal complication rates [15,17,20,21,22]. Endovascular treatment has become the preferred method for the management of VAPs in many cases, as it allows the pseudoaneurysm to be excluded from the arterial circulation, thus preventing its rupture and associated complications [17].

However, there are still some unanswered questions and ongoing research in this field. Firstly, there is a lack of consensus regarding the optimal endovascular technique (e.g., coil embolization, liquid embolic agents, stent grafts) as it depends on various factors, including the size, location, and morphology of the pseudoaneurysm, as well as the patient’s overall health condition [23]. Notably, the fluidic progression of Ethylene-Vinyl Alcohol (EVOH) Copolymer-Based Non-Adhesive Liquid Embolic Agents (NALEAs) facilitates the conveyance of liquid embolic agents through slender arteries, allowing them to effectively reach embolization targets positioned appreciably far from the microcatheter tip. Moreover, the preference for liquid embolic agents is recommended when performing vessel embolization for conditions involving vessel damage, such as VAPs [24,25,26]. Further studies are needed to evaluate the efficacy of different techniques and identify patient-specific factors that influence treatment selection. Secondly, there is a need for further investigation into the potential complications associated with endovascular repair of VAPs, such as stent graft migration, infection, endoleaks, visceral ischemia, and long-term vascular complications [17]. Understanding these risks and implementing strategies to minimize adverse events are essential for optimizing patient outcomes. Thirdly, there is a paucity of studies focused exclusively on reporting data regarding pseudoaneurysms, and the available studies exhibited limited sample sizes [15,27].

In this context, the purpose of the study was to retrospectively evaluate the efficacy and safety of endovascular treatment of visceral pseudoaneurysms using Ethylene-Vinyl Alcohol (EVOH) Copolymer-Based Non-Adhesive Liquid Embolic Agents (NALEAs). Data were collected exclusively on patients with VAPs, discarding cases of the endovascular treatment of VAAs.

## 2. Methodology

### 2.1. Study Design

**Figure 1:**
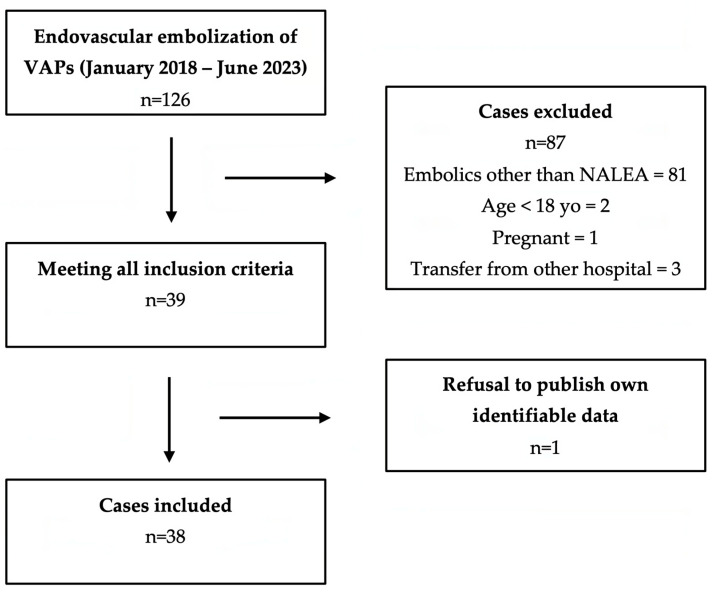
Flowchart of the selection of VAP cases treated with EVOH copolymer-based NALEAs. Abbreviations: *VAP*, Visceral Artery Pseudoaneurysm; *NALEA*: Non-Adhesive Liquid Embolic Agents; *EVOH*: Ethylene-Vinyl Alcohol.

Consecutive patients who underwent endovascular embolization for visceral artery pseudoaneurysms between January 2018 and June 2023 were retrospectively evaluated (Figure 1).

Inclusion criteria were: (I) transcatheter arterial embolization of visceral artery pseudoaneurysm performed with EVOH copolymer-based NALEA; (II) 18 years of age or older; (III) evaluation in a multidisciplinary team composed of surgeons, interventional radiologists, and anesthesiologists. The following exclusion criteria were applied: (I) pregnant or nursing females; (II) individuals with a platelet (PLT) count lower than 20,000 per microliter of blood, as per the recommendations of the Society of Interventional Radiology (SIR), who also declined blood component transfusion [28]; (III) International Normalized Ratio (INR) levels incompatible with femoral (>1.8) or radial (>2.2) artery access for low bleeding risk operations that require arterial access [28]; (IV) documented hypersensitivity to suitable embolic agents; (V) patients presenting at an emergency room of another hospital and subsequently transferred to our facilities.

Given the retrospective nature of the study, no ethical committee permission was required. The research adhered to the principles outlined in the Declaration of Helsinki. Prior to the endovascular procedure, written informed consent was obtained from each patient.

### 2.2. Treatment

The diagnostic workup included performing CT-angiography (CTA), which was necessary to make a diagnosis and plan endovascular intervention. Occasionally, critically ill patients with an already known diagnosis of VAP (e.g., by ultrasound examination) were transferred directly to the angiography room. A 24 h interventional radiology service was available at participating centers (Dulbecco University Hospital, Catanzaro, Italy; Circolo Hospital, Varese, Italy; Maggiore della Carità University Hospital, Novara, Italy; Mater Domini University Hospital, Catanzaro, Italy; Pugliese-Ciaccio Hospital, Catanzaro, Italy; San Timoteo Hospital, Termoli, Italy), so the first treatment option for VAPs was always endovascular embolization. An absolute contraindication for percutaneous interventions arises when preserving adequate blood flow to the target organ is of utmost importance and would be potentially compromised by the implementation of endovascular techniques, therefore, a surgical approach is indicated (e.g., hepatic artery nourishing a compromised liver and covered stent delivery not feasible) [29]. Patients symptomatic or with evidence at CTA of ruptured VAPs were treated on an urgent basis. Unruptured VAPs were treated as early as possible in the daytime [17].

**Figure 2:**
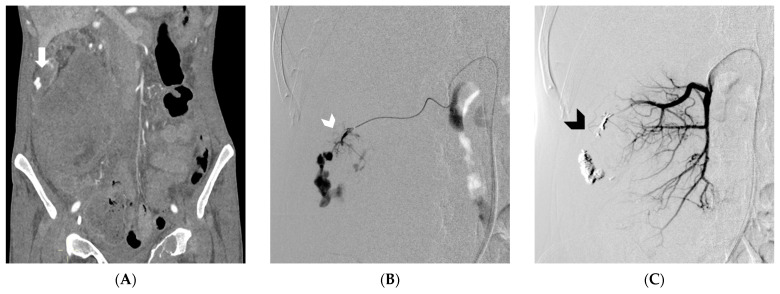
In Figure (**A**), Computed Tomography angiography reveals spontaneous retroperitoneal bleeding attributed to a ruptured pseudoaneurysm (indicated by the arrow) originating from a renal tumor. Figure (**B**) shows digital subtraction angiography, which confirms the presence of a ruptured pseudoaneurysm arising from a feeding artery of the tumor (white arrowhead). Lastly, Figure (**C**) displays digital subtraction angiography, illustrating the successful embolization achieved using an Ethylene-Vinyl Alcohol copolymer cast (indicated by the black arrowhead). (From Minici et al. doi: 10.3390/medicina59040710, by MDPI, Basel, Switzerland, licensed under CC BY 4.0).

**Figure 3:**
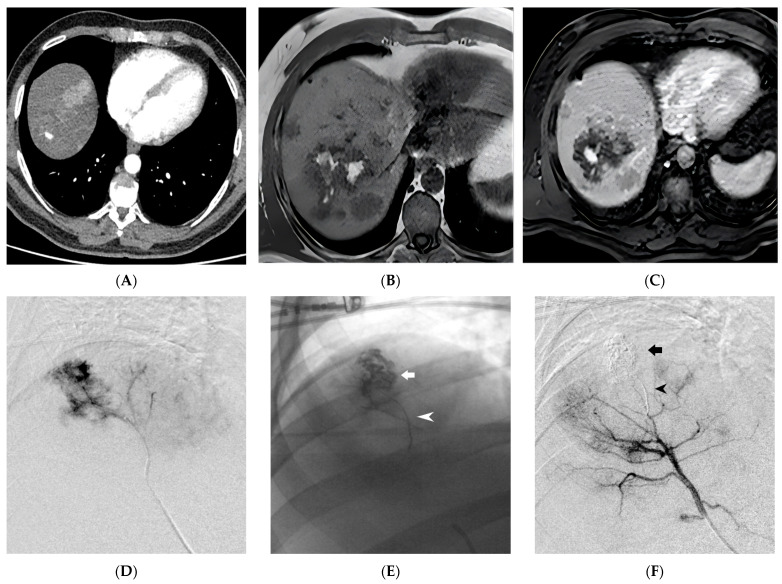
Ruptured Hepatocellular Carcinoma (HCC) within the hepatic parenchyma is depicted in the images. In the contrast-enhanced Computed Tomography, arterial phase, there is evidence of a 1 cm pseudoaneurysm within an HCC nodule (**A**). On the Magnetic Resonance Imaging (MRI) T1 Fast Field Echo In-Phase sequence, hyperintense material (blood) is observed within a large HCC nodule of the right lobe (**B**). Gadobenate dimeglumine (Gd-BOPTA)-enhanced MRI in the arterial phase confirms the presence of an intranodular contrast blush (**C**). Superselective digital subtraction angiography of the S7 hepatic artery branch shows contrast leakage into the ruptured HCC (**D**). Successful embolization of the HCC nodule (indicated by the arrow) and the parent artery (indicated by the arrowhead) was achieved using Ethylene-Vinyl Alcohol (EVOH) copolymer (**E**,**F**). (From Minici et al. doi: 10.3390/medicina59040710, by MDPI, Basel, Switzerland, licensed under CC BY 4.0).

**Figure 4:**
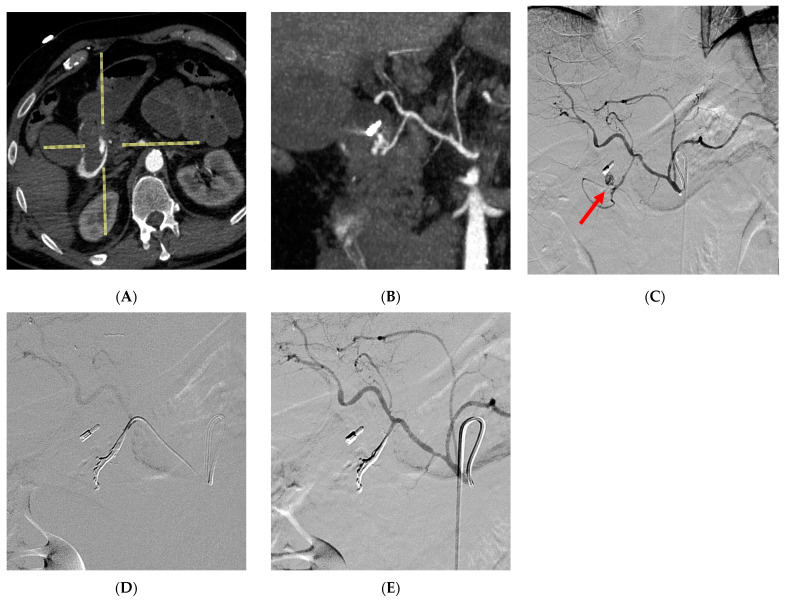
A young woman with a history of non-steroidal anti-inflammatory drug (NSAID) overuse experienced a sudden onset of hematemesis. During endoscopy, duodenal bleeding was observed but proved unresponsive to treatment. Advanced imaging techniques, including axial Computed Tomography scans (**A**) and multiplanar reconstruction (MPR) coronal reconstruction (**B**), revealed the presence of a pseudoaneurysm in the gastroduodenal artery. The celiac trunk was subsequently catheterized, confirming the presence of the pseudoaneurysm (arrow) close to the clip previously placed by the endoscopist at the site of bleeding (**C**). To address the condition, a superselective catheterization of the gastroduodenal artery was performed using a dimethyl sulfoxide (DMSO)-compatible microcatheter, followed by effective embolization using Ethylene-Vinyl Alcohol (EVOH) copolymer (**D**). The completion angiography confirmed the successful embolization, with the EVOH copolymer cast distributed along the gastroduodenal artery, ensuring no unintended embolizations to non-target areas (**E**).

In all patients undergoing angiography, a transfemoral retrograde approach was employed as first-choice vascular access. This approach offers the advantage of using a wide range of catheters with various shapes and sizes, enabling selective access to the splanchnic vessels. Heparinization was eventually performed to achieve an activated clotting time of 200 s. Following catheterization, angiography was performed by injecting iodinated contrast medium through the catheter to visualize the target vessel and identify any associated pathology. Subsequently, embolization materials or covered stent were delivered through the catheter under fluoroscopic guidance to address the pseudoaneurysm. Prior to embolization, ensuring the accurate positioning of the catheter tip is crucial to avoid unintentional non-target embolization. The endovascular procedure was conducted in specialized catheterization laboratories by a skilled interventional radiologist. The selection of the embolic agent was based on the personal preferences of the operator. Non-adhesive liquid embolic agents (NALEAs) were prepared following the provided instructions and administered under fluoroscopic guidance using a microcatheter compatible with dimethyl sulfoxide (DMSO) (Figure 2, Figure 3 and Figure 4). To prevent EVOH (ethylene-vinyl alcohol) copolymer precipitation, the dead space of the microcatheter, including the luer-lock hub at its proximal end, was filled with DMSO. The microcatheter was strictly single-use and not subjected to DMSO rinsing after the injection. By adhering to a controlled injection rate, ensuring it remains below the recommended threshold of 0.3 mL/min, the potential for reflux was minimized. This, in turn, reduced the risk of unintended embolization in non-target areas, thereby enhancing the precision and control over the delivery of embolic agents [25,30,31]. Evaluation of technical success and identification of any non-target embolization were carried out through postembolization angiography. Follow-up consisted of clinical examination at 1 and 6 months and of CTA at 6 months after embolization.

### 2.3. Outcomes and Definitions

The primary efficacy endpoint was to report the clinical success rate of endovascular treatment of VAPs using EVOH Copolymer-Based NALEAs. The secondary efficacy endpoint was to investigate the differences in technical success, clinical success, reintervention, and 30-day bleeding-related mortality rates between ruptured (rVAP) and unruptured (urVAP) VAPs. The primary safety endpoint of this study was determined to be the disparity in procedure-related complication rates between the two groups (ruptured vs. unruptured VAPs). End-organ infarction rate was evaluated as a secondary safety endpoint.

SIR reporting standards were used [32]. Technical success was defined by the effective exclusion of the VAP from circulation. The achievement of hemostasis in response to bleeding symptoms, prompting the need for TAE, established the fulfillment of clinical success. Time-to-embolization (TTE) was calculated as the time elapsed between diagnosis and successful embolization. VAP was defined as ruptured if active contrast extravasation was noted on CTA or angiography. Procedure-related complication rate included vascular access site complication (VASC) rate. The subgroup with coagulopathy was characterized using the criteria established by Loffroy et al., where coagulopathy was defined as having an International Normalized Ratio (INR) greater than 1.5, a partial thromboplastin time exceeding 45 s, or a platelet count below 80,000/mm^3^ [33]. Procedure-related complications were classified according to the 2017 SIR classification [34], the 2003 SIR classification [35], and the CIRSE classification [36].

### 2.4. Statistical Analysis

The data were recorded and organized in a Microsoft Excel spreadsheet (Microsoft Inc, Redmond, WA, USA), while statistical analyses were conducted based on the intention-to-treat principle using SPSS software (version 22 for Windows; SPSS Inc, Chicago, IL, USA) and R/R Studio software. The Modified Intention-To-Treat population, consisting of all randomized patients who underwent at least one embolization procedure, was utilized for the subsequent analyses [37,38]. Kolmogorov–Smirnov and Shapiro–Wilk tests were performed to verify the normality assumption of data. Categorical data are presented as frequencies (% value) [39]. Continuous data with a normal distribution are expressed as mean ± standard deviation, whereas continuous data that do not follow a normal distribution are presented as median (first to third quartile) [40,41]. Statistical differences in continuous data with a normal distribution were assessed using the unpaired Student *t*-test, while categorical data and continuous data without a normal distribution were evaluated using the Chi-squared/Fisher’s exact tests and the Mann–Whitney test, respectively [42,43,44]. A *p*-value of less than 0.05 was considered statistically significant for all the aforementioned tests.

## 3. Results

Endovascular embolization with EVOH-based NALEAs was performed in 38 patients with VAPs. The population was divided into two groups based on whether the pseudoaneurysm ruptured (rVAP group, 17 subjects) or was intact (urVAP group, 21 subjects). The urVAP and rVAP groups did not differ statistically in age, gender, BMI, renal and coagulation function, baseline hemoglobin, antiplatelet and anticoagulant therapy, and CTA execution. Individuals with rVAP showed more frequent symptoms and hemodynamic instability than individuals with urVAP. Interestingly, the rVAP group had a significantly shorter maximum pseudoaneurysm diameter than the urVAP group (3.3 vs. 4.4 cm; *p* = 0.005). Demographic and clinical data are reported in Table 1.

The most common etiologies were trauma, iatrogenic causes (e.g., biopsy, percutaneous nephrolithotomy (PCNL), surgery), and medical conditions (e.g., pancreatitis, cholecystitis, cancer, abscess). The distribution of pseudoaneurysm locations did not significantly differ between the groups (*p* = 0.830). The most common sites were splenic, hepatic, and renal arteries. Four unruptured (one common hepatic artery, one proper hepatic artery, two intrahepatic) and three ruptured (one proper hepatic artery, two intrahepatic) VAPs of hepatic arteries were treated. The majority of patients with unruptured pseudoaneurysms underwent treatment as soon as possible, while patients with ruptured pseudoaneurysms had more urgent treatment (*p* < 0.001). The distribution of EVOH viscosity did not show significant differences between the groups (*p* = 0.449). There were no significant differences in time and radiation-related variables (CT-to-groin time, procedure time, CT-to-embolization time, fluoroscopy time, cumulative air kerma (CAK), and dose area product (DAP)) between the groups. Procedure data are summarized in Table 2.

The technical success rate was 100% for all patients, indicating that the endovascular treatment was successful in achieving the desired outcome. The clinical success rate, indicating resolution of symptoms, was high in both groups (92.1% overall), with no significant differences between the unruptured and ruptured groups (*p* = 0.679). The incidence of rebleeding was low in both groups, with no significant differences observed (*p* = 0.819). The need for blood transfusion was significantly higher in the rVAP group compared to the urVAP group (*p* < 0.001). The occurrence of non-target embolization was low overall (2.6%), related to only one case of non-occluding reflux in the right hepatic artery without clinical sequelae. Seven patients (18.4%) experienced procedure-related complications, related to one case of non-target embolization, four splenic abscesses due to end-organ infarction (two cases managed with antibiotic therapy, while the other two with percutaneous drainage), one case of femoral pseudoaneurysm treated with US-guided thrombin injection and another case of femoral pseudoaneurysm managed with prolonged groin compression. The rates of procedure-related complications, end-organ infarction, and vascular access-site complications did not significantly differ between the unruptured and ruptured VAP groups (*p* > 0.05). The mortality rate due to bleeding within 30 days was low overall (linked to a single case of multiple organ dysfunction syndrome in a patient with major trauma), with no significant differences between the urVAP and rVAP groups (*p* = 0.362). Details are reported in Table 3.

## 4. Discussion

The outcomes of our investigation into endovascular embolization of VAPs utilizing EVOH-based NALEAs can be succinctly outlined as follows:

- 100% technical success and 92.1% clinical success rates;
- 18.4% procedure-related complication and 2.6% 30-day bleeding-related mortality rates;
- no substantial disparities in efficacy and safety outcomes between VAPs that have ruptured and those that have not.

Visceral pseudoaneurysms form a heterogeneous category due to their occurrence in diverse arterial locations and their association with a range of etiologies [17,29]. The etiology of VAPs recognizes a few main causes, including trauma, iatrogenic procedures (e.g., biopsies, percutaneous nephrolithotomy, surgery, radiation therapy, etc.), and medical conditions (e.g., acute pancreatitis, tumors, abscesses, etc.) [15,16]. The majority of the studies available in the literature include a mixed population comprising both VAAs and VAPs, thus introducing an additional source of data heterogeneity given their distinct pathological conditions [15]. In addition, the assessment of outcomes in endovascular treatment is further complicated by the presence of studies in which various endovascular techniques were employed, but the outcomes were not differentiated based on the specific technique used [29,45,46]. It would be desirable to have outcome data specific to each technique in order to conduct comparative evaluations among homogeneous patient groups according to the site and etiology of the VAP.

Endovascular treatment aims to effectively exclude the VAP from the systemic circulation [47]. Nagaraja et al. conducted one of the few comparative assessments between surgery and angioembolization for the treatment of hepatic artery pseudoaneurysms, concluding that both techniques are effective. However, they found that endovascular treatment offers faster hemostasis, reduced transfusion requirements, and shorter hospital stays [48]. The choice of endovascular technique and embolic agent relies on several factors, including the size of the pseudoaneurysm sac and neck, the expendability of the parent artery, the presence of terminal or collateral circulation, the feasibility of superselective catheterization of the pseudoaneurysm considering vessel tortuosity and hostile anatomy, the availability of adequate landing zones for a covered stent, the patient’s coagulation status, and the operator’s preferences [23,49,50].

Deliberate parent artery sacrifice is generally considered in cases of end-arteries (e.g., intraparenchymal renal pseudoaneurysm) or when the presence of collateral circulations minimizes the risk of end-organ ischemia. Fascinatingly, the study conducted by Nagaraja et al. provided evidence that the occlusion of the common hepatic artery is generally well-tolerated when treating VAPs in patients with preserved liver function. This favorable tolerance is attributed to the phenomenon of flow reversal in the gastroduodenal artery and the hypertrophy of branches originating from the inferior phrenic artery [48,51]. In the presence of collateral circulation (e.g., splenic, hepatic, and gastroduodenal VAPs), the sandwich embolization of afferent and efferent vessels is essential to achieve effective embolization, thus preventing backdoor bleeding [47]. Conversely, in the case of end-arteries (e.g., renal circulation), proximal embolization of the afferent artery alone proved to be effective [52,53]. Liquid embolics may allow the avoidance of direct negotiation of the pseudoaneurysm sac; however, they are associated with a risk of non-target embolization. In such situations, in addition to adjusting the density of the liquid embolic, it is possible to employ the sandwich technique by pre-embolizing the outflow with coils to minimize the risk of unintentional leakage of the liquid embolic [22,47,54,55].

When intentional parent artery sacrifice is not indicated, it is necessary to exclude the VAP from the circulation while preserving the patency of the parent artery [23,47]. The task can be accomplished through selective embolization of the pseudoaneurysm sac using coils, vascular plugs, or liquid embolics such as NALEA or n-butyl cyanoacrylate (NBCA) [56]. Unlike coils, liquid embolics can avoid exerting radial force on the sac, but they carry a higher risk of reflux and non-target embolization [22,54]. In some cases of wide neck, stent-assisted or balloon-remodeling techniques can be employed to provide a scaffold for safer delivery of the embolic agent [47,57]. Finally, in the presence of adequate landing zones, the pseudoaneurysm can be excluded while preserving the patency of the parent artery through the deployment of a covered stent [58,59]. The successful placement of stent grafts is often impeded by the small size or tortuous nature of numerous visceral arteries, leading to a restricted range of stent graft options available [60]. Recently, the use of multilayered flow-diverting stents has emerged in the treatment of true visceral aneurysms due to their ability to induce flow stagnation and subsequent thrombosis within the sac while preserving the patency of the parent artery and any collateral branches originating from the segment covered by the stent [61]. However, evidence regarding their use in visceral pseudoaneurysms is limited, as thrombosis requires time to occur, whereas the risk of imminent rupture remains substantial [23,62,63].

This study aims to mitigate the data heterogeneity prevalent in the literature by presenting the outcomes of endovascular treatment specifically for VAPs managed by embolization with EVOH Copolymer-Based NALEAs. While the outcomes of this treatment in the study are comparable to other research, assessing the results is complicated due to the frequent case mix in previous reports (i.e., VAAs/VAPs, endovascular/surgical treatments, parent artery sacrifice/preservation, various embolic agents). Limited evidence exists on endovascular treatment using EVOH Copolymer-Based NALEAs, often involving mixed reports on true and false aneurysms [64]. Previous reports on the coil embolization technique pointed out interesting results, such as Ghoneim’s 80% success rate in renal arterial pseudoaneurysms [27] and Regus’ 100% technical success rate in diverse VAPs [16]. Other studies, like Zabicki’s investigation of pancreatitis-related pseudoaneurysms [45], Balderi’s examination of endovascular repair of VAAs/VAPs [46], Fankhauser’s large cohort analysis [29], and Laganà’s technical results for VAPs [65], contribute to the understanding of the effectiveness of endovascular treatment. Roberts’ research emphasizes a high success rate even in cases of bleeding ruptured VAPs [66]. Therefore, endovascular treatment of both ruptured and non-ruptured visceral pseudoaneurysms using EVOH copolymer-based non-adhesive liquid embolic agents (NALEAs) has comparable efficacy to other embolic agents, as demonstrated by this literature review, and can be a valuable tool, especially in certain clinical scenarios where the unique characteristics of NALEAs offer particular advantages.

Firstly, the unique flow characteristics resembling molten magma facilitate the transportation of the embolic agent through narrow arteries, enabling successful embolization of targets located at considerable distances from the microcatheter tip [24,26]. In contrast, the efficacy of coil embolization is diminished when dealing with small-caliber vessels that are challenging to catheterize, and it is less effective in controlling backdoor bleeding stemming from collateral circulation if the efferent vessels are not embolized [67,68]. Secondly, EVOH-based NALEAs ensure a secure, swift, and efficient embolization process through polymerization, thereby accomplishing mechanical embolization without necessitating coagulation activation. This property is particularly advantageous in patients with coagulation disorders, as supported by previous studies [24,26,69]. Intriguingly, coil embolization exhibits reduced efficacy in individuals with coagulopathy, while liquid embolics do not appear to encounter this limitation [33,70,71,72,73,74,75]. Thirdly, in the study conducted by Khalil et al., it was recommended that EVOH be prioritized for embolization procedures involving compromised blood vessels. This preference stems from the fact that EVOH is capable of deployment without exerting radial pressure on the vessel walls, in contrast to coils and vascular plugs that have the potential to cause rupture in fragile vessels [25].

Repeated endovascular treatment, surgery, and percutaneous or endoscopic embolization are the main options if the first-line endovascular therapy fails [66]. If endovascular management is the first-line therapy, surgery will be needed in less than 5% of cases [66]. Roberts et al. reported a 17% reintervention rate [66]. The underlying causes for the endovascular treatment failure were diverse, yet they predominantly fell into two discernible categories: either the endovascular access to the VAP was unsuccessful or the initial attempts at hemorrhage control failed. In instances where endovascular access proved unsuccessful, alternative methods such as percutaneous embolization or surgical repair were employed to attain effective control. Conversely, in cases where initial attempts at hemorrhage control failed, it was deemed reasonable to make repeated efforts utilizing endovascular management, as this approach yielded satisfactory results in half of such instances. A major cause of bleeding control failure is ineffective hemostasis with coils [66]. In our study, we observed a very low reintervention rate, which may be due to the effectiveness of NALEAs in patients with coagulopathy. In this study, endovascular embolization using EVOH Copolymer-Based NALEAs has been proven to be a safe treatment for both ruptured and unruptured pseudoaneurysms. Other reports on the endovascular management of VAPs have shown comparable data. Ghoneim et al. reported a 13.3% complication rate for endovascular management of 15 renal arterial pseudoaneurysms using coil post-partial nephrectomy [27]. Regus et al. recorded a 14.3% complication rate in their cohort of 7 VAPs treated with coils or surgery, with one bleeding case [16]. Zabicki et al. found a 13.3% complication rate in their investigation of pancreatitis-related pseudoaneurysms using various techniques [45]. End-organ ischemia is one of the most feared complications, but evidence agrees that its practical consequences are often minimal. Balderi observed 50% splenic ischemia and 75% renal infarction but minimal practical consequences, with only 3.2% requiring surgery [46]. Fankhauser reported 13.5% hepatic or splenic infarction, with a 7.3% VASC rate [29]. Laganà reported a 27.6% complication rate with seven splenic infarction cases out of eight complications [65]. Roberts highlighted a 6.1% complication rate, demonstrating safety in bleeding ruptured VAPs [66]. Furthermore, the safety results of our report, including vascular access site complications, align with previous studies conducted on endovascular treatments and transcatheter arterial embolization [76,77,78,79,80,81,82,83,84,85,86,87,88,89,90,91,92,93,94,95,96,97,98,99,100,101,102,103,104,105,106,107,108,109,110,111]. Finally, this retrospective multicenter study provides further evidence supporting the safety and efficacy of endovascular embolization using EVOH copolymer-based non-adhesive liquid embolic agents (NALEAs) for the management of visceral pseudoaneurysms. These findings also extend to patients with ruptured visceral pseudoaneurysms, highlighting the suitability of this treatment approach in such cases.

However, it is important to acknowledge several limitations associated with EVOH-based non-adhesive liquid embolic agents (NALEAs). Firstly, it is necessary to shake the mixture containing EVOH and tantalum powder vigorously for a duration of 20 min to make the embolic agent ready for use. Consequently, this may limit its application in time-sensitive scenarios [78,112,113]. At our institute, we have successfully tackled this concern by instructing staff to initiate shaking of the vials with a Vortex shaking mixer upon activating the catheterization lab for an angioembolization [114]. Secondly, dimethyl sulfoxide (DMSO) requires the use of a DMSO-compatible microcatheter and exhibits toxicity towards blood vessels, resulting in vasospasm, pain, and the occurrence of atypical reflexes like the trigeminocardiac reflex [55,115,116,117,118]. Thirdly, the addition of tantalum powder to EVOH-based NALEAs enhances radiopacity, facilitating visibility during the embolization procedure; however, it can introduce beam hardening artifacts in subsequent CT examinations. To address this issue, both Onyx and Squid offer reduced tantalum formulations known as Onyx L and Squid LD, respectively, which minimize the occurrence of beam hardening artifacts [119,120].

Some limitations of the study should be noted. The extensive length of the follow-up period instills confidence that our investigation has captured the majority of recurrences or treatment-related complications. However, it is important to acknowledge that the possibility of unreported complications, recurrences, or even fatalities cannot be completely ruled out. Notably, Guillon et al. observed no cases of long-term recurrence in their own study [121]. The implementation of a multicenter registry would allow the collection of data prospectively and the ability to carry out comparative evaluations for homogeneous groups taking into account etiology, comorbidities, endovascular technique, and embolic agent used. The retrospective nature of the analysis also weakens the study as some cases may have been missed, although we believe this hypothesis unlikely given the research carried out using the radiological electronic database present in all participating centers.

## 5. Conclusions

In conclusion, this is the first multicenter investigation evaluating the feasibility of endovascular embolization of visceral pseudoaneurysms using EVOH copolymer-based non-adhesive liquid embolic agents (NALEAs). Our data demonstrate that both ruptured and unruptured visceral pseudoaneurysms can be effectively and safely treated with NALEA-based endovascular embolization, with outcomes similar to those of other embolic agents. We suggest considering the use of NALEAs, particularly in specific clinical cases that highlight their advantages. These cases may include patients with coagulopathy, particularly fragile vessels, and embolization targets that are located at a considerable distance from the microcatheter tip and are otherwise difficult to reach. Nevertheless, further larger comparative studies with long-term follow-ups would be desirable to confirm these preliminary data.

## Figures and Tables

**Table 1:** Baseline demographic and clinical variables of the sample: descriptive analyses according to ruptured or unruptured status of the pseudoaneurysm.

Variables	All Patients (n = 38)			
		Unruptured VAP (n = 21)	Ruptured VAP (n = 17)	*p*-Value
Age (years)	55.7 (±23.9)	53.7 (±25.5)	58.2 (±22.3)	0.547
Sex (M/F)	11 (28.9%)/27 (71.1%)	7 (33.3%)/14 (66.7%)	4 (23.5%)/13 (76.5%)	0.721
BMI	25.7 (±4)	25.8 (±4)	25.6 (±4.1)	0.918
eGFR (mL/min)	71.4 (±22.2)	75.8 (±19.8)	66.1 (±24.5)	0.290
INR	1.26 (±0.3)	1.26 (±0.3)	1.25 (±0.3)	0.965
aPTT (s)	40.2 (±5.5)	40 (±5.5)	40.5 (±5.4)	0.768
Platelet count (No. ×10^3^/μL)	341 (±91.8)	335.4 (±98.2)	348 (±85.6)	0.692
Coagulopathy	9 (23.7%)	6 (28.6%)	3 (17.6%)	0.476
Baseline Hemoglobin (g/dL)	7.7 (±0.8)	7.4 (±0.5)	8 (±1)	0.093
Antiplatelet therapy	9 (23.7%)	5 (23.8%)	4 (23.5%)	1
Anticoagulant therapy	11 (28.9%)	5 (23.8%)	6 (35.3%)	0.491
Hemodynamic instability	7 (18.4%)	0 (0%)	7 (41.2%)	0.002
Symptomatic Pseudoaneurysm	24 (63.2%)	7 (33.3%)	17 (100%)	<0.001
CT-angiography execution	37 (97.4%)	21 (100%)	16 (94.1%)	0.447
VAP max diameter (cm)	3.9 (±1.2)	4.4 (±1.2)	3.3 (±0.9)	0.005

Abbreviations: *VAP*: Visceral Artery Pseudoaneurysm; *BMI*: Body Mass Index; *eGFR*: Estimated Glomerular Filtration Rate; *INR*: International Normalized Ratio; *aPTT*: activated partial thromboplastin time; *CT*: Computed Tomography.

**Table 2:** Procedure data.

Variables	All Patients (n = 38)			
		Unruptured VAP (n = 21)	Ruptured VAP (n = 17)	*p*-Value
Etiology - trauma - iatrogenic (e.g., biopsy, PCNL, surgery) - medical conditions (e.g., pancreatitis, cancer, abscess) - others	16 (42.1%) 9 (23.7%) 12 (31.6%) 1 (2.6%)	8 (38.1%) 6 (28.6%) 6 (28.6%) 1 (4.8%)	8 (47.1%) 3 (17.6%) 6 (35.3%) 0 (0%)	0.660
Site of the pseudoaneurysm - splenic artery - hepatic artery - gastric artery - gastroduodenal artery - gastroepiploic artery - pancreaticoduodenal artery - renal artery	13 (34.2%) 7 (18.4%) 1 (2.6%) 4 (10.5%) 3 (7.9%) 4 (10.5%) 6 (15.8%)	7 (33.3%) 4 (19%) 0 (0%) 3 (14.3%) 1 (4.8%) 2 (9.5%) 4 (19%)	6 (35.3%) 3 (17.6%) 1 (5.9%) 1 (5.9%) 2 (11.8%) 2 (11.8%) 2 (11.8%)	0.830
Endovascular treatment timing - as soon as possible - urgent	14 (36.8%) 24 (63.2%)	14 (66.7%) 7 (33.3%)	0 (0%) 17 (100%)	<0.001
- Proximal embolization (e.g., proper hepatic artery) - Distal embolization (e.g., intrasplenic, intrahepatic)	18 (47.4%) 20 (52.6%)	11 (52.4%) 10 (47.6%)	7 (41.2%) 10 (58.8%)	0.532
EVOH viscosity (centiStokes) - 12 - 18 - 20 - 34	2 (5.3%) 6 (15.8%) 7 (18.4%) 23 (60.5%)	1 (4.8%) 4 (19%) 2 (9.5%) 14 (66.7%)	1 (5.9%) 2 (11.8%) 5 (29.4%) 9 (52.9%)	0.449
Intraoperative contrast medium (mL)	33.6 (±12)	30.9 (±11.9)	36.9 (±11.6)	0.100
Volume of contrast to creatinine clearance ratio	0.58 (±0.54)	0.45 (±0.29)	0.74 (±0.73)	0.064
Vascular access site - Femoral - Radial - Brachial	34 (89.5%) 2 (5.3%) 2 (5.3%)	18 (85.7%) 1 (4.8%) 2 (9.5%)	16 (94.1%) 1 (5.9%) 0 (0%)	0.424
Sheath diameter - 4F - 5F - ≥6F	7 (18.4%) 27 (71.1%) 4 (10.5%)	4 (19%) 16 (76.2%) 1 (4.8%)	3 (17.6%) 11 (64.7%) 3 (17.6%)	0.435
CT-to-groin time (min)	143.8 (±179)	199.2 (±225.9)	75.3 (±35.6)	0.150
Procedure time (min)	39.7 (±10.3)	41 (±11.6)	38.1 (±8.5)	0.444
CT-to-embolization time (min)	186.4 (±182.4)	245.9 (±229)	112.8 (±31.5)	0.177
Fluoroscopy time (min)	14.6 (±5.9)	15.1 (±6)	14 (±5.8)	0.744
Cumulative air kerma (mGy)	218 (±68.2)	206.3 (±68.1)	232.3 (±67.6)	0.246
Dose area product (Gy/cm^2^)	34.7 (±10.1)	33.7 (±10)	35.9 (±10.3)	0.537

Abbreviations: *VAP*: Visceral Artery Pseudoaneurysm; *PCNL*: percutaneous nephrolithotomy; *EVOH*: ethylene-vinyl alcohol; *CT*: Computed Tomography.

**Table 3:** Outcomes data.

Variables	All Patients (n = 38)			
		Unruptured VAP (n = 21)	Ruptured VAP (n = 17)	*p*-Value
Technical success	38 (100%)	21 (100%)	17 (100%)	1
Clinical success	35 (92.1%)	19 (90.5%)	16 (94.1%)	0.679
Rebleeding	5 (13.2%)	3 (14.3%)	2 (11.8%)	0.819
Repeated XA - None - Same bleeding site - Different bleeding site	33 (86.8%) 2 (5.3%) 3 (7.9%)	18 (85.7%) 1 (4.8%) 2 (9.5%)	15 (88.2%) 1 (5.9%) 1 (5.9%)	0.911
Imaging follow-up modality - Computed Tomography Angiography - Magnetic Resonance Angiography - Ultrasound	34 (89.5%) 1 (2.6%) 3 (7.9%)	18 (85.7%) 0 (0%) 3 (14.3%)	16 (94.1%) 1 (5.9%) 0 (0%)	0.154
Vascular access site hemostasis - Manual compression - Vascular closure device	19 (50%) 19 (50%)	11 (52.4%) 10 (47.6%)	8 (47.1%) 9 (52.9%)	0.744
Units of packed red blood cells transfused per patient	1.6 (±2.1)	0.6 (±0.7)	2.9 (±2.5)	<0.001
Non-target embolization	1 (2.6%)	1 (4.8%)	0 (0%)	0.362
Procedure-related complication Rate	7 (18.4%)	4 (19%)	3 (17.6%)	0.912
End-organ infarction Rate	4 (10.5%)	2 (9.5%)	2 (11.8%)	0.823
Vascular access-site complication Rate	2 (5.3%)	1 (4.8%)	1 (5.9%)	0.878
Procedure-related Complications (SIR classification) - None - Minor (grade 1–2) - Major (grade 3–4–5)	31 (81.6%) 7 (18.4%) 0 (0%)	17 (81%) 4 (19%) 0 (0%)	14 (82.4%) 3 (17.6%) 0 (0%)	0.912
Procedure-related Complications (CIRSE classification) - None - Grade 2 - Grade 3	31 (81.6%) 1 (2.6%) 6 (15.8%)	17 (81%) 1 (4.8%) 3 (14.3%)	14 (82.4%) 0 (0%) 3 (17.6%)	0.644
30-day bleeding-related mortality	1 (2.6%)	1 (4.8%)	0 (0%)	0.362

Abbreviations: *VAP*: Visceral Artery Pseudoaneurysm; *XA*: X-ray angiography; *SIR*: Society of Interventional Radiology; *CIRSE*: Cardiovascular and Interventional Society of Europe.

## Data Availability

The data presented in this study are available on request from the corresponding author. The data are not publicly available due to privacy issues.

## Abbreviations

APTT: activated partial thromboplastin time; CKD: chronic kidney disease; CIRSE: Cardiovascular and Interventional Society of Europe; CTA: CT-angiography; DAP: dose-area product; DSA: digital subtraction angiography; eGFR: estimated Glomerular Filtration Rate; EVOH: ethylene-vinyl alcohol; ICU: intensive care unit; INR: international normalized ratio; MRA: MR-angiography; NALEA: non-adhesive liquid embolic agent; NBCA: N-butyl cyanoacrylate; PCNL: percutaneous nephrolithotomy; PRBC: packed red blood cells; PT: prothrombin time; PVA: polyvinyl alcohol; SIR: Society of Interventional Radiology; TAE: transcatheter arterial embolization; TTE: time-to-embolization; US: ultrasound; VAA: visceral artery aneurysm; VAP: visceral artery pseudoaneurysm; VASCs: vascular access site complications; XA: X-ray angiography.
